# Role of pancreatic ductal adenocarcinoma risk factors in intraductal papillary mucinous neoplasm progression

**DOI:** 10.3389/fonc.2023.1172606

**Published:** 2023-06-06

**Authors:** Manuel Gentiluomo, Chiara Corradi, Paolo Giorgio Arcidiacono, Stefano Crippa, Massimo Falconi, Giulio Belfiori, Riccardo Farinella, Laura Apadula, Gaetano Lauri, Niccolò Bina, Cosmeri Rizzato, Federico Canzian, Luca Morelli, Gabriele Capurso, Daniele Campa

**Affiliations:** ^1^ Department of Biology, University of Pisa, Pisa, Italy; ^2^ Pancreato-Biliary Endoscopy and Endosonography Division, Pancreas Translational and Clinical Research Center, Istituti di Ricovero e Cura a Carattere Scientifico (IRCCS) San Raffaele Scientific Institute, Milan, Italy; ^3^ Unit of Pancreatic Surgery, Pancreas Translational and Clinical Research Center, San Raffaele Scientific Institute, Vita-Salute University, Milan, Italy; ^4^ Department of Translational Research and of New Technologies in Medicine and Surgery, University of Pisa, Pisa, Italy; ^5^ Genomic Epidemiology Group, German Cancer Research Center (DKFZ), Heidelberg, Germany; ^6^ General Surgery Unit, Department of Translational Research and New Technologies in Medicine and Surgery, University of Pisa, Pisa, Italy; ^7^ Digestive and Liver Disease Unit, Sant’Andrea University Hospital, Faculty of Medicine and Psychology, Sapienza University of Rome, Rome, Italy

**Keywords:** pancreatic ductal adenocarcinoma (PADC), intraductal papillary mucinous neoplasms (IPMN), single nucleotide polymorphism (SNP), association study, polygenic score (PGS)

## Abstract

**Introduction:**

Pancreatic ductal adenocarcinoma (PDAC) is lethal due to its late diagnosis and lack of successful treatments. A possible strategy to reduce its death burden is prevention. Intraductal papillary mucinous neoplasms (IPMNs) are precursors of PDAC. It is difficult to estimate the incidence of IPMNs because they are asymptomatic. Two recent studies reported pancreatic cysts in 3% and 13% of scanned subjects. The possibility of identifying a subgroup of IPMN patients with a higher probability of progression into cancer could be instrumental in increasing the survival rate. In this study, genetic and non-genetic PDAC risk factors were tested in a group of IPMN patients under surveillance.

**Methods:**

A retrospective study was conducted on 354 IPMN patients enrolled in two Italian centres with an average follow-up of 64 months. With the use of DNA extracted from blood, collected at IPMN diagnosis, all patients were genotyped for 30 known PDAC risk loci. The polymorphisms were analysed individually and grouped in an unweighted polygenic score (PGS) in relation to IPMN progression. The ABO blood group and non-genetic PDAC risk factors were also analysed. IPMN progression was defined based on the development of worrisome features and/or high-risk stigmata during follow-up.

**Results:**

Two genetic variants (rs1517037 and rs10094872) showed suggestive associations with an increment of IPMN progression. After correction for multiple testing, using the Bonferroni correction, none of the variants showed a statistically significant association. However, associations were observed for the non-genetic variables, such as smoking status, comparing heavy smokers with light smokers (HR = 3.81, 95% 1.43–10.09, *p* = 0.007), and obesity (HR = 2.46, 95% CI 1.22–4.95, *p* = 0.012).

**Conclusion:**

In conclusion, this study is the first attempt to investigate the presence of shared genetic background between PDAC risk and IPMN progression; however, the results suggest that the 30 established PDAC susceptibility polymorphisms are not associated with clinical IPMN progression in a sample of 354 patients. However, we observed indications of cigarette smoking and body mass index (BMI) involvement in IPMN progression. The biological mechanism that could link these two risk factors to progression could be chronic inflammation, of which both smoking and obesity are strong promoters.

## 1 Introduction

Pancreatic ductal adenocarcinoma (PDAC) is a hard-to-treat cancer (HTTC), and it is projected to become the second most common cancer in terms of mortality by 2030 in the United States (1). In the past decade, only modest improvement has been obtained in terms of survival time, which, only recently, approached 11% at 5 years after diagnosis in the United States (2). This meagre survival is principally due to the late diagnosis when, usually, the tumour is in an advanced stage. Early diagnosis is challenging for the lack of specific symptoms and a comprehensive understanding of the aetiology of the disease (3). Compared to more common cancers, only a small number of environmental/lifestyle factors have been established (4, 5). Our knowledge of the genetics of PDAC is also relatively limited, with approximately 30 susceptibility loci identified (6–14). Considering that there is no successful treatment apart from surgery, prevention remains the best option to decrease the death toll for this disease. Up to 8%–10% of the patients undergoing abdominal imaging harbour unsuspected pancreatic cysts in the general population, the majority of which are intraductal papillary mucinous neoplasms (IPMNs), well-known precursors of PDAC (15, 16). The precise incidence of IPMN is challenging to estimate due to the asymptomatic nature of this condition, which makes the diagnosis difficult, leading to an underestimation of the incidence. Two studies that carried out a series of computed tomography scans in subjects without history or symptoms associated with pancreatic diseases have reported pancreatic cysts in 3% and 13% of scanned subjects (17, 18). The diagnosis of high-risk IPMNs offers the unique opportunity to treat a direct precursor of PDAC. IPMNs are common, with an estimated prevalence of 3%–6% in the general population that rises to 10% in the elderly (19–21). Surgery is the only curative approach for IPMN with suspicion of malignancy, but pancreatectomy is still associated with high morbidity and mortality rates (22). Moreover, a considerable fraction of IPMN patients do not progress to invasive cancer (23, 24). There are significant differences in the incidence rate of pancreatic malignancy among the different IPMN forms. Branch-duct (BD) IPMNs have a progression rate to pancreatic cancer of 3.3% after 5 years from initial diagnosis, which rises to 15% after 15 years. Compared to BD, main-duct (MD) and mixed-type (MT) IPMNs have higher progression rates of 6%–45% and 60%–90%, respectively (25–29). Current guidelines identify high-risk stigmata (HRSs) and worrisome features (WFs) as indications for surgical resection (30). Although WFs and HRSs are useful indicators of clinical progression, they are not necessarily markers of progression towards cancer. Nevertheless, a recent study reported that patients with at least a WF at diagnosis are more prone to progress to malignancy if they develop additional WFs or HRSs during surveillance (31). The identification of endogenous (e.g., genetic variability) and exogenous (e.g., lifestyle factors such as smoking and alcohol consumption) markers of progression is of the uttermost importance since it could improve the stratification of patients according to their risk of developing PDAC. Several lines of evidence suggest that lifestyle and genetic factors play a role in transforming IPMN into PDAC (32–36). Since carcinogenesis is a complex and multifactorial process, the only viable way to predict individuals’ risks is to analyse them with a comprehensive approach (33, 34). Additionally, it has been proposed that PDAC risk factors may play a role in IPMN progression. For example, non-O blood group carriers have an increased risk of developing PDAC and have been suggested to have a higher chance of acquiring WFs and/or HRSs (34). Therefore, in this study, with the aim of finding progression markers, we have tested 30 single-nucleotide polymorphisms (SNPs), the ABO blood group, and five non-genetic PDAC risk factors in a group of 354 IPMN patients under follow-up, recruited in two high-volume Italian centres.

## 2 Materials and methods

### 2.1 Study population

The retrospective study population consisted of 354 subjects with a diagnosis of IPMNs enrolled by two Italian centres: 273 individuals from San Raffaele Research Hospital (Milan, Italy) and 81 from Ospedale Cisanello-University of Pisa (Pisa, Italy). The diagnosis of IPMN was ascertained by the observation of magnetic resonance imaging with magnetic resonance cholangiopancreatography and/or endoscopic ultrasonography. The inclusion criteria were as follows: a) age of >18 years, b) will and can cooperate, and c) a certain diagnosis of IPMN, with a follow-up of at least 12 months. Exclusion criteria from the study were the absence of follow-up and indication for surgery at diagnosis. WFs were defined as a) cyst size ≥3 cm, b) enhancing mural nodule <5 mm, c) thickened enhanced cyst walls, d) main pancreatic duct (MPD) size between 5 and 9 mm, e) abrupt change in MPD diameter with distal pancreatic atrophy, f) lymphadenopathy, g) increased serum level of CA19-9, h) cyst growth rate >5 mm/2 years, and i) acute pancreatitis. HRS was defined as a) obstructive jaundice caused by the IPMN, b) enhancing mural nodule ≥5 mm, and c) MPD ≥ 10 mm. WFs and HRSs at diagnosis were defined according to the International Association of Pancreatology guidelines (30). A small subgroup (46 from San Raffaele and 10 from Pisa) already showed at least one WF at diagnosis and was followed up like all the other enrolled subjects.

An aliquot of whole peripheral blood was collected at IPMN diagnosis to conduct genetic analyses. The study obtained institutional review board (IRB) approval in both the enrolling centres: San Raffaele Research Hospital, protocol number 133/2016 (for recording clinical information) protocol BIOGASTRO 2011, amendment 6/11/2017 (for blood sample biobanking and biomarker investigation); Cisanello Hospital-University of Pisa, the ethical committee was the “Comitato Etico Regionale per la Sperimentazione Clinica della Toscana sezione Area Vasta Nord Ovest”, protocol code “IPMN-PDAC transition”. The study population details are shown in **Table 1**.

**Table 1:** Description of the study population.

	No WFs at diagnosis		All IPMNs	
	No. of patients with progression (%)	No. of patients with no progression (%)	No. of patients with progression (%)	No. of patients with no progression (%)
Branch-duct IPMN	78 (26)	204 (68)	91 (26)	223 (63)
Mixed-type IPMN	12 (4)	3 (1)	22 (6)	9 (3)
Main-duct IPMN	1 (0.34)	0	7 (2)	2 (0.6)
Age, median (IQR), years	68 (61.0–68.0)	66 (60.0–72.0)	67 (60.0–73.0)	67 (60.0–73.0)
Follow-up time, median (IQR), months	67 (37.0–104.0)	59.99 (32.3–90.03)	60 (31.5–91.17)	61.7 (31.6–92.05)
Sex				
Male	43 (14)	73 (24)	60 (17)	89 (25)
Female	48 (16)	134 (45)	60 (17)	145 (41)
BMI				
<18	7 (2)	11 (4)	7 (2)	12 (3)
18–25.9	45 (15)	126 (42)	59 (16)	138 (39)
26–29.9	26 (9)	53 (18)	34 (10)	62 (18)
≥30	106 (36)	11 (4)	13 (4)	17 (5)
Not available	3 (1)	7 (2)	7 (1)	5 (1)
Smoking				
No (never)	61 (20)	135 (45)	76 (22)	149 (42)
Yes	30 (10)	72 (24)	44 (12)	85 (24)
Alcohol consumption				
No (never)	53 (18)	118 (40)	67 (19)	134 (38)
Yes	38 (13)	89 (30)	53 (15)	100 (28)
Type 2 diabetes				
No	78 (26)	187 (63)	103 (29)	210 (60)
Yes	13 (4)	20 (7)	17 (5)	24 (7)
Cyst size at diagnosis (mm)				
<15	32 (11)	125 (42)	40 (11)	133 (38)
15.1–29.9	59 (20)	82 (28)	59 (17)	84 (24)
≥30			21 (6)	17 (5)

Age and progression time are expressed in years and months, respectively. Progression means patients without WFs or HRSs and patients with WFs at diagnosis who developed the first or an additional WF or HRS during the follow-up. No progression means the no-development of new WFs or HRSs during the follow-up.

BMI, body mass index (calculated as weight in kilograms divided by height in meters squared); IQR, interquartile range; WFs, worrisome features; HRSs, high-risk stigmata; IPMN, intraductal papillary mucinous neoplasm.

### 2.2 Study outcomes

Four outcomes of interest were defined: I) development of the first WF and/or HRS for patients who did not present any at diagnosis, II) development of an additional WF or HRS for patients showing one at diagnosis, III) development of more than two WFs or HRSs for patients who did not present any at diagnosis, and IV) development of HRSs in naïve patients.

### 2.3 SNP selection

The 30 known PDAC risk SNPs identified so far were selected to generate a polygenic score (PGS) representative of the risk of developing PDAC. In detail, 28 SNPs, identified through genome-wide association studies (GWASs), were selected (12, 37–41). Furthermore, two SNPs identified in two candidate gene association studies were added (42, 43). In addition, two SNPs were used to infer the ABO blood group, specifically rs505922, which discriminates O from non-O, and rs8176746, which distinguishes between A and B alleles (41, 44). The selected SNPs and their association with PDAC risk are shown in **Supplementary Table 1**. This specific PGS was designed and validated in two studies on PDAC susceptibility (45, 46).

### 2.4 Non-genetic PDAC risk factors

At diagnosis, in addition to sex and age, data on smoking expressed as smoking status (smokers/non-smokers) and divided into classes based on the number of cigarettes smoked per day (light smoker, a smoker who reports consuming between 1 and 10 cigarettes per day; moderate smoker, 11–20 cigarettes per day; heavy smoker, 21 cigarettes or more per day), diabetes status, (yes/no), body mass index (BMI) (usual BMI before diagnosis) were collected. BMI was also categorised into four classes: underweight (BMI < 18.5), healthy weight (BMI >18.5 to <25), overweight (BMI 25 to <30), and obese (BMI 30 or higher). Finally, a family history of pancreatic cancer was reported as a binary variable, considering the presence of pancreatic cancer cases in at least one first-degree relative.

### 2.5 Genotyping

Genomic DNA of IPMN patients was extracted from whole blood using the QIAamp DNA Kit (QIAGEN, CA, USA) and genotyped in 384-well plates with TaqMan technology (Thermo Fisher Applied Biosystems, Waltham, MA, USA). In addition to the samples, no-template controls and duplicated samples (8%), used for quality control purposes, were included on each plate. The endpoint fluorescence reading of the plates and the assignment of the genotype were performed using QuantStudio 5 Real-Time PCR system (Thermo Fisher Applied Biosystems, Waltham, MA, USA) and QuantStudio software. The frequencies of the 32 SNPs genotyped in IPMN were tested for deviation from the Hardy–Weinberg equilibrium using Pearson’s chi-square test.

### 2.6 Statistical analysis and score computation

Association with progression was calculated as hazard ratio (HR) using the Cox regression multivariate analysis adjusted for age and sex. The same analyses were performed for the four defined outcomes. The different types of IPMN were compared, using branch-duct IPMNs as reference. The PGS was calculated for all the subjects with a genotyping call rate (CR) higher than 80%, summing the number of PDAC risk alleles, thus generating a score with a value within 0 to 60 and adding the value associated with the ABO blood group, with a value of 0 for the OO group and 1 for the other blood groups. To compensate for the missing genotypes in the subjects with a CR lower than 100%, the PGS was scaled by dividing PGS by the subject CR. The PGS computation is described in detail elsewhere (45). Applying the Bonferroni correction (dividing the threshold value of 0.05 by the number of tests: 30 SNPs and 10 non-genetic factors, for a total of 40 independent tests), we considered the *p*-value = 0.0013 as the threshold for statistical significance.

## 3 Results

Of 354 patients with a median age of 67 years (IQR 63.0–70.0 years), during an average follow-up time of 64 months, 91 patients developed the first WF and/or HRS, while 29 patients developed an additional one. Of the 354 patients, 205 were female, and 149 were male. BD IPMNs were the most common (314), followed by MT (31) and MD IPMNs (9). The information on BMI was collected for 342 subjects. Among these, 197 (58%) had normal weight, 96 (28%) were overweight, 30 (9%) were obese, and 19 (6%) were underweight. A total of 225 patients (64%) were never smokers, while 129 (36%) were current smokers. In addition, data on alcohol consumption were collected for 354 patients; of these, 201 (57%) were non-drinkers. The study population characteristics are summarised in **Table 1**.

### 3.1 Genetic association analysis

A total of 30 SNPs were analysed individually and combined in a PGS in relation to IPMN clinical progression. All SNPs were in Hardy–Weinberg equilibrium, and the average genotyping call rate was 99%.

A statistically significant association (*p* ≤ 0.05) was observed in the model that included only the patient without WFs at diagnosis, with the T allele of 18q21.32-rs1517037 increasing the probability of IPMN progression (HR = 1.55, 95% CI 1.06–2.29, *p* = 0.026). A similar result was observed in the analyses carried out on patients without WFs at diagnosis who developed more than two WFs or HRSs during follow-up. The results are shown in **Supplementary Table 1**.

Moreover, in the model that included the patients who showed WFs at diagnosis, the A allele of the 8q24.21-rs10094872 was associated with increased progression probability (HR = 1.39, 95% CI 1.02–1.89, *p* = 0.043). After correction for multiple testing (using the Bonferroni correction), none of the variants showed a statistically significant association.

The PGS was not associated with IPMN clinical progression (*p* > 0.05). Also, the individual blood group was not associated with progression. The results of the association analysis of SNPs and blood groups are shown in **Table 2**.

**Table 2:** Results of the association analysis between ABO blood group, PDAC susceptibility loci analysed individually and combined in a PGS, and the risk of IPMN progression.

Number of subjects (progressed/non-progressed)			No WFs at diagnosis		All IPMNs	
			298 (91/207)	-	354 (234/120)	-
***Blood group***			**HR (95% CI)**	***p***	**HR (95% CI)**	***p***
Non-O blood group *vs.* O group			0.89 (0.59–1.35)	0.581	0.97 (0.67–1.39)	0.857
A blood group *vs.* O group			0.93 (0.60–1.45)	0.761	0.97 (0.66–1.43)	0.896
AB blood group *vs.* O group			1.24 (0.49–3.15)	0.646	1.22 (0.52–2.85)	0.642
B groups *vs.* O group			0.54 (0.21–1.37)	0.193	0.80 (0.38–1.68)	0.556
***Genetic variant***	**RA**	**EA**	**HR (95% CI)**	***p***	**HR (95% CI)**	***p***
rs7046076	t	c	0.89 (0.64–1.26)	0.515	0.96 (0.71–1.29)	0.781
rs2035875	g	a	1.07 (0.77–1.47)	0.694	1.11 (0.83–1.47)	0.479
rs13303010	a	g	1.1 (0.73–1.67)	0.639	1.12 (0.77–1.61)	0.555
rs2736100	a	c	0.94 (0.70–1.26)	0.689	0.97 (0.75–1.25)	0.821
rs351365	c	t	0.87 (0.60–1.26)	0.459	0.85 (0.62–1.18)	0.345
rs2816938	a	t	0.86 (0.61–1.21)	0.388	0.82 (0.60–1.10)	0.185
rs3790844	a	g	0.97 (0.66–1.45)	0.901	0.98 (0.68–1.40)	0.899
rs1486134	t	g	0.77 (0.54–1.10)	0.152	0.75 (0.54–1.04)	0.082
rs9854771	g	a	0.84 (0.61–1.16)	0.292	0.92 (0.71–1.20)	0.561
rs2853677	a	g	0.99 (0.74–1.34)	0.964	1.05 (0.82–1.36)	0.681
rs2736098	t	c	0.92 (0.66–1.29)	0.641	0.96 (0.72–1.29)	0.79
rs35226131	c	t	0.63 (0.31–1.28)	0.200	0.72 (0.44–1.20)	0.214
rs401681	c	t	1.10 (0.81–1.49)	0.561	1.11 (0.86–1.44)	0.422
rs17688601	c	a	0.94 (0.69–1.28)	0.697	0.94 (0.72–1.23)	0.671
rs73328514	a	t	0.95 (0.59–1.53)	0.840	1.03 (0.67–1.56)	0.905
rs6971499	t	c	0.95 (0.57–1.58)	0.851	0.95 (0.61–1.48)	0.835
rs172310	c	a	1.15 (0.85–1.57)	0.369	1.19 (0.92–1.55)	0.189
rs2941471	a	g	0.98 (0.72–1.34)	0.914	0.92 (0.70–1.21)	0.548
rs10094872	t	a	1.30 (0.92–1.85)	0.141	1.39 (1.02–1.89)	0.035
rs1561927	t	c	0.89 (0.62–1.29)	0.543	1.03 (0.75–1.42)	0.846
rs10991043	t	c	0.98 (0.72–1.33)	0.874	1.05 (0.80–1.37)	0.739
rs7310409	g	a	0.94 (0.69–1.29)	0.716	1.02 (0.78–1.33)	0.895
rs9581943	g	a	1.04 (0.79–1.36)	0.794	0.97 (0.77–1.24)	0.829
rs9543325	t	c	1.19 (0.86–1.64)	0.298	1.13 (0.86–1.49)	0.382
rs8028529	t	c	0.87 (0.56–1.35)	0.523	0.84 (0.57–1.24)	0.385
rs7190458	g	a	0.53 (0.21–1.33)	0.175	0.69 (0.34–1.40)	0.305
rs4795218	g	a	0.91 (0.63–1.32)	0.624	1.05 (0.76–1.44)	0.779
rs11655237	c	t	0.82 (0.50–1.35)	0.435	0.88 (0.58–1.33)	0.539
rs1517037	c	t	1.54 (1.05–2.25)	0.027	1.23 (0.89–1.70)	0.209
rs16986825	c	t	0.81 (0.54–1.21)	0.299	1.04 (0.75–1.42)	0.829
rs8176746	c	a	0.69 (0.36–1.34)	0.272	0.88 (0.50–1.54)	0.652
rs505922	t	c	0.98 (0.71–1.34)	0.882	0.98 (0.74–1.28)	0.856
PGS						
2nd *vs.* 1st quintile			1.25 (0.67–2.31)	0.480	1.29 (0.74–2.23)	0.366
3rd *vs.* 1st quintile			1.50 (0.79–2.85)	0.219	1.38 (0.78–2.44)	0.271
4th *vs.* 1st quintile			1.04 (0.56–1.94)	0.902	1.01 (0.56–1.82)	0.977
5th *vs.* 1st quintile			0.90 (0.41–2.00)	0.802	1.18 (0.66–2.09)	0.579

All the analyses were adjusted by sex and age.

PGS, polygenic risk score; RA, reference allele; EA, effect allele; PDAC, pancreatic ductal adenocarcinoma; PGS, polygenic score; IPMN, intraductal papillary mucinous neoplasm; WFs, worrisome features.

### 3.2 Non-genetic factors

Several statistically significant associations (*p* ≤ 0.05) were observed for the non-genetic variables. The number of cigarettes smoked per day, analysed by dividing the smokers into light, moderate, and heavy smokers, was associated with IPMN progression, HR = 3.81 (95% 1.43–10.09, *p* = 0.007), in the model that included IPMN patients without WFs at diagnosis, and HR = 2.98 (95% CI 1.26–7.04, *p* = 0.013) analysing all the IPMN patients. Instead, the smoking status (ever *vs.* never) was not associated with progression.

Furthermore, obese patients (BMI > 30 kg/m^2^) showed an increased risk of clinical progression compared to healthy weight patients (BMI 18–25 kg/m^2^), with an HR = 2.46 (95% CI 1.22–4.95, *p* = 0.01) in the model with the patients without WFs at diagnosis, and HR = 2.03 (95% CI 1.17–3.52, *p* = 0.012) in the model that included all the patients.

Analysing the risk of clinical progression of branch-duct, mixed-type, and main-duct IPMNs, the MD, compared to the BD, showed an increased probability of progression, in particular in the model that included just the patients without WFs at diagnosis, HR = 10.17 (95% CI 1.34–77.13, *p* = 0.025), a trend confirmed in the more inclusive model, HR = 3.52 (95% CI 1.60–7.74, *p* = 0.002). The mixed-type IPMN also showed an increment of the probability of progression but was less evident than main-duct IPMN, HR = 2.37 (95% CI 1.24–4.55, *p* = 0.009) and HR = 1.69 (95% CI 1.04–2.76, *p* = 0.035), in the model with all patients.

Finally, diabetes and a family history of pancreatic cancer were not associated with clinical IPMN progression. Sex (HR = 1.27, 95% CI 1.01–2.38, *p* = 0.035), age (HR = 1.03, 95% CI 1.01–1.06, *p* = 0.011), the size of the largest cyst (HR = 1.07, 95% CI 1.04–1.10, *p* = 3 × 10^−5^), and pancreatitis (HR = 3.02, 95% CI 1.40–6.53, *p* = 0.005) were associated with IPMN progression (**Table 3**). After correction for multiple testing, none of the variables showed a statistically significant association, except for the size of the largest cyst. In the analyses conducted in patients without WFs at diagnosis who developed more than two WFs or HRSs during follow-up, the association with the size of the cyst was confirmed, HR = 1.20 (95% CI 1.11–1.31, *p* = 1.53 × 10^−5^). In this analysis, an association with chronic pancreatitis was also observed, HR = 3.40 (95% CI 1.06–10.88, *p* = 0.039). Results are shown in **Supplementary Table 2**.

**Table 3:** Results of the association analysis between the known PDAC risk factors and the IPMN progression.

*Characteristic*	No WFs at diagnosis				All IPMNs			
	HR (95% CI)	*p*	No. of prog.	No. of no prog.	HR (95% CI)	*p*	No. of prog.	No. of no prog.
**Age (years)**	1.03 (1.01–1.06)	**0.011**	91	207	1.03 (1.01–1.05)	**0.008**	120	234
**Sex (male vs. female)**	1.57 (1.01–2.38)	**0.035**	43 vs. 48	73 vs. 134	1.43 (0.99–2.05)	0.052	60 vs. 60	89 vs. 145
**Smoking (smokers vs. non-smokers)**	0.84 (0.54–1.32)	0.453	30 vs. 61	72 vs. 135	0.95 (0.65–1.39)	0.785	44 vs. 76	85 vs. 149
**Moderate smokers vs. light smokers**	0.90 (0.36–2.26)	0.83	8 vs. 12	24 vs. 41	1.18 (0.59–2.35)	0.638	17 vs. 16	30 vs. 44
**Heavy smokers vs. light smokers**	3.81 (1.43–10.09)	**0.007**	8 vs. 12	6 vs. 41	2.98 (1.26–7.04)	**0.013**	9 vs. 16	8 vs. 44
**Alcohol consumption (drinkers vs. non-drinkers)**	0.97 (0.61–1.52)	0.86	38 vs. 53	89 vs. 118	1.07 (0.73–1.57)	0.725	53 vs. 67	100 vs. 134
**Underweight (<18 kg/m^2^)**	2.07 (0.91–4.70)	0.084	7 vs. 45	11 vs. 126	1.59 (0.71–3.54)	0.261	7 vs. 59	12 vs. 139
**Overweight (26–30 kg/m^2^)**	1.29 (0.80–2.11)	0.299	26 vs. 45	53 vs. 126	1.25 (0.81–1.92)	0.311	34 vs. 59	62 vs. 139
**Obesity and class 3 obesity (>30.1 kg/m^2^)**	2.46 (1.22–4.95)	**0.012**	10 vs. 45	11 vs. 126	2.03 (1.17–3.52)	**0.012**	17 vs. 59	13 vs. 139
**Family history of pancreatic cancer**	1.27 (0.66–2.46)	0.479	10 vs. 81	21 vs. 186	1.36 (0.77–2.38)	0.29	14 vs. 106	22 vs. 212
**Main duct vs. branch duct**	10.17 (1.34–77.13)	**0.025**	1 vs. 78	0 vs. 204	3.52 (160–7.74)	**0.002**	7 vs. 91	1 vs. 224
**Mixed type vs. branch duct**	2.37 (1.24–4.55)	**0.009**	12 vs. 78	3 vs. 204	1.69 (1.04–2.76)	**0.035**	22 vs. 91	9 vs. 224
**Type 2 diabetes**	1.39 (0.77–2.52)	0.27	13 vs. 78	20 vs. 187	1.18 (0.70–1.98)	0.536	17 vs. 103	24 vs. 210
*WFs and HRSs*								
**Cyst size (mm)**	1.07 (1.04–1.10)	**3.00 × 10^−5^**	207	91	1.02 (1.00–1.04)	**0.016**	120	234
**Cyst size 15–29.9 vs. <14.9 mm**	1.81 (1.15–2.84)	**0.009**	59 vs. 32	82 vs. 125	1.67 (1.11–2.50)	**0.013**	59 vs. 40	84 vs. 133
**Cyst size >30 vs. <14.9 mm**	*-*	*-*	*-*	*-*	2.26 (1.31–3.94)	**0.004**	21 vs. 40	17 vs. 133
**Pancreatitis (acute and chronic pancreatitis)**	1.24 (0.60–2.50)	0.454	9 vs. 82	17 vs. 190	1.60 (1.00–2.57)	**0.048**	23 vs. 97	21 vs. 213
**Acute pancreatitis**	*-*	*-*	*-*	*-*	3.02 (1.40–6.53)	**0.005**	8 vs. 99	2 vs. 216
**Chronic pancreatitis**	1.13 (0.51–2.48)	0.757	7 vs. 84	12 vs. 193	1.22 (0.67–2.22)	0.508	13 vs. 99	14 vs. 216

The analyses of BMI are all performed by comparing each category with normal weight (18.5–25 kg/m^2^). The association level of variables, cyst size >30 mm, and acute pancreatitis were not calculated in the model “No WFs at diagnosis” because there are two worrisome features. No. of prog. = the number of subjects who showed an IPMN progression; the non-continuous variables are reported as the number of analysed subjects vs. the number of subjects of the reference category. No. of no prog. = the number of subjects who did not show an IPMN progression. All the analyses were adjusted by sex and age.

PDAC, pancreatic ductal adenocarcinoma; IPMN, intraductal papillary mucinous neoplasm; WFs, worrisome features; BMI, body mass index.

Text in bold indicates associations with p≤0.05.

## 4 Discussion

Considering that PDAC is an HTTC, detection at non-malignant stages is, at the moment, the best strategy to lower the death burden associated with the disease. A viable approach is to focus on high-risk individuals, among which IPMN patients are a large proportion. The estimated IPMN progression rate to PDAC ranges from 3%–15% for BD IPMNs to 40%–90% for MD IPMNs (25–28, 47–50). Therefore, identifying IPMNs that will evolve into PDAC will be instrumental for detecting PDAC cases during the early stages and will also limit the overtreatment of patients who do not require surgery. Since some PDAC risk factors such as BMI and heavy cigarette smoking have been suggested to play a role in the development of WFs and HRSs in IPMN patients (34), in this study, we analysed several PDAC risk factors (30 PDAC susceptibility SNPs, the ABO blood group, and five non-genetic PDAC risk factors) in relation to IPMN progression.

According to the current knowledge, our data confirmed the greater tendency to clinical progression of MD IPMN when compared with BD IPMN. Furthermore, we observed that the MD has 10 times more possibility to progress than BD, HR = 10.17 (1.34–77.33, *p* = 0.025).

### 4.1 Genetic background and IPMN progression

We observed two SNPs associated with IPMN progression with a *p*-value lower than 0.05. The T allele of the 18q21.32-rs1517037 and the A allele of 8q24.21-rs10094872 were associated with IPMN progression. rs1517037 showed similar results in the model that considered patients without WFs at diagnosis who developed more than two WFs or HRSs during follow-up, making rs1517037 a potential candidate for future studies on IPMN progression. This SNP is an intergenic variant near the gastrin-releasing peptide (*GRP*) gene, and the C allele is associated with PDAC risk. This variant is also associated with several traits related to PDAC risks, such as BMI, diabetes, and pancreas volume (51–54). The T allele of rs10094872 is associated with PDAC risk; this variant is situated near the *MYC* proto-oncogene. *MYC* plays a role in cell cycle progression, apoptosis, and cellular transformation, and mutations in this gene are frequently observed in numerous human cancers (55–60). Although these associations are intriguing, they are not statistically significant when applying multiple testing corrections. Additionally, the alleles associated with increased PDAC risk are associated with a lower probability of IPMN progression in our study. Therefore, these associations are probably due to statistical fluctuation. Thus, the results suggest the absence of a shared genetic background between PDAC risk and IPMN progression. This conclusion agrees with our previous results, which identified a germline variant associated with clinical IPMN progression that is not associated with PDAC risk (33).

We did not observe a statistically significant association between the ABO blood group and IPMN progression, which further supports the different genetic predispositions for IPMN progression and PDAC risk. The role of the non-O blood group in IPMN progression in non-resected IPMN patients was investigated only in one study by Capurso and colleagues, which observed an increased risk of progression in the A blood group compared to O subjects (34). A possible explanation for the difference between the results of the two studies could be ascribed to the different sample sizes since the present study includes four times more patients than the other. Three studies investigating the role of the ABO blood group in IPMN-resected patients reported heterogeneous results (61–63). Zelga and colleagues, analysing 819 resected IPMN patients, reported a significant predominance of the non-O blood group compared with the O blood group in patients with malignant IPMNs (63). Instead, Poruk and colleagues reported that O blood group individuals had a higher probability of having high-grade dysplasia in a sample of 777 resected IPMNs (61). Finally, Hamada and colleagues analysed 1,815 Japanese patients and found that A or B blood group individuals have a higher risk of developing PDAC when compared with those in the O blood group (62). Therefore, the role of the ABO blood group in IPMN progression remains controversial also for resected patients.

### 4.2 Non-genetic factors and IPMN progression

Our results suggest that clinical IPMN progression and PDAC risk share, at least partially, the same non-genetic risk factors. We observed an association between two established PDAC risk factors, cigarette smoking and BMI, with IPMN progression. Cigarette smoking was already observed in association with IPMN progression; Carr et al. reported that smoking may accelerate the development of IPMN and the subsequent transformation into invasive cancer (64). The role of smoking was also reported in a Japanese population, in agreement with our observation in Europeans (65). In pancreatic cancer, the molecular mechanisms of cigarette smoking are currently unknown; however, a synergic role of nicotine metabolites and rare mutations (i.e., *KRAS* mutations) has been proposed. Higher nicotine retention was observed in some specific organs, including the pancreas, with high nicotine concentrations and its derivatives in pancreatic tissue and pancreatic juice (66–68). In a study by Prokopczyk and colleagues, smokers and non-smokers were compared, reporting clear evidence of higher nicotine derivative accumulation in the pancreatic juice of smokers, which was up to seven times higher than in non-smokers (67). Therefore, the mechanism that induces pancreatic cancer could also be involved in IPMN progression. Exposure to smoking causes the activation of immune cells, which induces the production of proinflammatory factors with consequent recruitment of immune cells, including T cells, macrophages, dendritic cells, and neutrophils. The cascade of immune cell production promoted by cigarette smoking may result in chronic inflammation.

We also observed an increased progression rate in obese patients compared with healthy-weight patients, in agreement with a previous study, which suggests the association of obesity with an increased frequency of malignancy in BD IPMN (69). The association was partially confirmed by the analyses conducted considering the patients who develop new HRSs.

Conversely, the analyses on the patients who developed two or more WFs did not support the association of cigarette smoking and BMI with IPMN progression. These latter analyses might represent the group more likely to progress towards cancer but were conducted on a limited number of subjects, reducing the power to detect an association. The accumulation of body fat has been associated with several diseases, including pancreatic cancer (70). In obese individuals, adipose cells around the pancreas produce an inflammatory environment (71), and the adipocytes associated with pancreatic cancer cells secrete higher than normal adipokines, chemokines, proinflammatory cytokines, and growth factor levels, which may accelerate the neoplastic process (72). All these observations suggest a central role of the chronic inflammatory process, driven by the accumulation of body fat, in the evolution of IPMNs towards pancreatic cancer. Chronic inflammation results in an immunosuppressive tumour microenvironment, which may promote tumour development through dysregulated cell division, fast proliferation, and prolonged cell survival (73, 74).

### 4.3 Study limitations

A possible limitation of the study is the fact that all patients are under surveillance with no final diagnosis following surgery, and thus malignancy (high grade and invasive) is unknown for most of the individuals. Therefore, the associations that we observed are with clinical progression, and a validation in a set of IPMN patients who underwent resection and for which final pathology has been determined is warranted. Nevertheless, Marchegiani and colleagues showed that patients with at least a WF at diagnosis have an increased probability to evolve into high-grade dysplasia if they acquire additional WFs or HRSs (31). This evidence highlights the importance of identifying markers of the progression of new WFs and HRSs.

### 4.4 Conclusion

In conclusion, the results of our study show that the 30 established PDAC susceptibility polymorphisms are not associated with clinical IPMN progression in a sample of 354 patients either individually or combined in a score. However, the results suggest proinflammatory habits such as smoking or patient characteristics such as obesity as common factors among PDAC risk and IPMN progression.

## Data availability statement

The datasets presented in this article are not readily available because of legal and privacy restrictions. Requests to access the datasets should be directed to the corresponding author.

## Ethics statement

The study obtained IRB approval in both the enrolling centres. S. Raffaele Research Hospital: protocol number 133/2016 (for recording clinical information) protocol BIOGASTRO 2011, amendment 6/11/2017 (for blood samples biobanking and for biomarkers investigation). Cisanello Hospital-University of Pisa: the ethical committee was the “Comitato Etico Regionale per la Sperimentazione Clinica della Toscana sezione Area Vasta Nord Ovest”, protocol code “IPMN-PDAC transition”. The patients/participants provided their written informed consent to participate in this study.

## Author contributions

DC and GC conceived the study. MG and CC performed experimental work. MG and CC performed data analysis. All other authors contributed to the collection of samples and data. MG and DC drafted the manuscript, and all other authors took part in its critical revision. GC and DC share the last authorship. All authors contributed to the article and approved the submitted version.
