# Supplementary material for: Role of pancreatic ductal adenocarcinoma risk factors in intraductal papillary mucinous neoplasm progression
Source: Front Oncol. 2023 Jun 6;13:1172606. doi: 10.3389/fonc.2023.1172606 (PMC10280811; doi:10.3389/fonc.2023.1172606)
Supplement: Supplementary file 1 [file Table_1.docx]

**Supplementary table 1.** Results of the association analysis between the genetic components associated with the risk of developing PDAC (blood group, 30 known risk loci and PGS) and the risk of IPMN progression defined with more stringed criteria.

|  |  |  | **>2WF/HRS** |  | **HRS** |  |
| --- | --- | --- | --- | --- | --- | --- |
| ***Blood group*** |  |  | HR (95% CI) | P | HR (95% CI) | P |
| **Non-O blood group vs O group** |  |  | 0.57 (0.21-1.54) | 0.266 | 0.63 (0.16-2.55) | 0.52 |
| **A blood groups vs O group** |  |  | 0.93 (0.60-1.45) | 0.36 | 1.50 (0.44-5.09) | 0.516 |
| **AB blood group vs O group** |  |  | - | - | 0.48 (0.09-2.43) | 0.375 |
| **B groups vs O group** |  |  | 0.69 (0.14-3.40) | 0.651 | 0.55 (0.11-2.81) | 0.475 |
| **Genetic variant** | RA | EA |  |  |  |  |
| rs7046076 | t | c | 0.62 (0.27-1.43) | 0.261 | 0.51 (0.22-1.18) | 0.116 |
| rs2035875 | g | a | 1.02 (0.50-2.08) | 0.948 | 0.76 (0.37-1.56) | 0.459 |
| rs13303010 | a | g | 0.87 (0.29-2.55) | 0.795 | 1.82 (0.80-4.17) | 0.155 |
| rs2736100 | a | c | 0.87 (0.43-1.80) | 0.722 | 1.23 (0.63-2.41) | 0.546 |
| rs351365 | c | t | 0.79 (0.33-1.87) | 0.597 | 0.84 (0.37-1.91) | 0.681 |
| rs2816938 | a | t | 0.88 (0.40-1.99) | 0.769 | 0.79 (0.36-1.73) | 0.56 |
| rs3790844 | a | g | 0.57 (1.17-1.87) | 0.350 | 0.50 (0.15-1.62) | 0.248 |
| rs1486134 | t | g | 0.60 (0.24-1.48) | 0.268 | 0.50 (0.21-1.22) | 0.13 |
| rs9854771 | g | a | 0.61 (0.27-1.38) | 0.236 | 0.58 (0.27-1.24) | 0.161 |
| rs2853677 | a | g | 1.08 (0.54-2.18) | 0.828 | 1.16 (0.59-2.26) | 0.665 |
| rs2736098 | t | c | 0.55 (0.28-1.11) | 0.094 | 0.66 (0.33-1.33) | 0.246 |
| rs35226131 | c | t |  |  | - | - |
| rs401681 | c | t | 0.57 (0.25-1.29) | 0.174 | 0.80 (0.38-1.64) | 0.541 |
| rs17688601 | c | a | 0.61 (0.27-1.38) | 0.235 | 1.15 (0.59-2.24) | 0.675 |
| rs73328514 | a | t | 1.18 (0.51-2.74) | 0.702 | 1.33 (0.60-2.96) | 0.478 |
| rs6971499 | t | c | 0.74 (0.21-2.54) | 0.628 | 0.72 (0.21-2.43) | 0.593 |
| rs172310 | c | a | 1.02 (0.49-2.13) | 0.964 | 1.71 (0.88-3.34) | 0.114 |
| rs2941471 | a | g | 0.91 (0.45-1.84) | 0.793 | 0.96 (0.49-1.88) | 0.916 |
| rs10094872 | t | a | 1.59 (0.72-3.45) | 0.253 | 1.67 (0.78-3.57) | 0.185 |
| rs1561927 | t | c | 1.13 (0.50-2.55) | 0.764 | 0.95 (0.42-2.15) | 0.898 |
| rs10991043 | t | c | 0.77 (0.36-1.63) | 0.49 | 0.73 (0.35-1.51) | 0.398 |
| rs7310409 | g | a | 0.94 (0.44-1.95) | 0.863 | 0.68 (0.33-1.42) | 0.305 |
| rs9581943 | g | a | 1.66 (0.87-3.20) | 0.125 | 1.18 (0.63-2.25) | 0.599 |
| rs9543325 | t | c | 1.10 (0.51-2.39) | 0.81 | 1.11 (0.54-2.29) | 0.772 |
| rs8028529 | t | c | 1.41 (0.64-3.10) | 0.396 | 1.53 (0.71-3.32) | 0.286 |
| rs7190458 | g | a | 0.49 (0.06-3.76) | 0.495 | 1.12 (0.25-5.02) | 0.880 |
| rs4795218 | g | a | 0.49 (0.18-1.38) | 0.179 | 0.86 (0.38-1.93) | 0.717 |
| rs11655237 | c | t | 0.26 (0.04-1.93) | 0.192 | 0.23 (0.03-1.71) | 0.152 |
| rs1517037 | c | t | 2.48 (1.10-5.56) | **0.028** | 2.8 (1.41-5.62) | **0.003** |
| rs16986825 | c | t | 0.55 (0.18-1.68) | 0.298 | 0.78 (0.31-1.98) | 0.61 |
| rs8176746 | c | a | 0.58 (0.13-2.65) | 0.48 | 0.29 (0.04-2.24) | 0.238 |
| rs505922 | t | c | 0.73 (0.32-1.61) | 0.429 | 1.03 (0.50-2.14) | 0.929 |
| **PGS** |  |  |  |  |  |  |
| **2^nd^ vs 1^st^ quintile** |  |  | 0.57 (0.10-3.10) | 0.512 | 0.80 (0.31-2.10) | 0.654 |
| **3^rd^ vs 1^st^ quintile** |  |  | 2.17 (0.63-7.46) | 0.219 | 1.02 (0.38-2.76) | 0.967 |
| **4^th^ vs 1^st^ quintile** |  |  | 0.61 (0.13-2.73) | 0.513 | - | - |
| **5^th^ vs 1^st^ quintile** |  |  | - | - | 0.43 (0.53-3.50) | 0.433 |

**>2WF/HRS** = analyses, no progression versus those with >2 WR/HRS features; **HRS** = analyses, no progression versus HRS alone. **RA**: reference allele, **EA**: effect allele. All the analyses were adjusted by sex and age. PGS: polygenic risk score.
