# Supplementary material for: Role of pancreatic ductal adenocarcinoma risk factors in intraductal papillary mucinous neoplasm progression
Source: Front Oncol. 2023 Jun 6;13:1172606. doi: 10.3389/fonc.2023.1172606 (PMC10280811; doi:10.3389/fonc.2023.1172606)
Supplement: Supplementary file 2 [file Table_2.docx]

**Supplementary table 2.** Results of the association analysis between the known PDAC risk factors and the risk of IPMN progression defined with more stringed criteria.

|  | **>2 WF/HRS** | | | | **HRS** | | | |
| --- | --- | --- | --- | --- | --- | --- | --- | --- |
| ***Characteristic*** | **HR (95% CI)** | **P** | N of pro | N of no prog | **HR (95% CI)** | **P** | **N of pro** | **N of no prog** |
| **Age (years)** | 1.10 (1.02-1.19) | **0.015** | 16 | 207 | 1.03 (0.97-1.09) | 0.317 | 18 | 207 |
| **Sex (males vs females)** | 2.22 (0.83-5.99) | 0.112 | 9 vs 7 | 73 vs 134 | 1.89 (0.75-4.82) | 0.178 | 9 vs 9 | 73 vs 134 |
| **Smoking (smokers vs non-smokers)** | 0.61 (0.19-1.94) | 0.398 | 4 vs 12 | 72 vs 135 | 0.54 (0.18-1.69) | 0.294 | 4 vs 14 | 72 vs 135 |
| **Moderate smokers vs Light smokers** | - | - | 0 vs 3 | 6 vs 41 | **-** | **-** | 0 vs 3 | 6 vs 41 |
| **Heavy smokers vs Light smoker** | 0.26 (0.03-2.60) | 0.252 | 1 vs 3 | 24 vs 41 | 0.38 (0.4-3.90) | 0.417 | 1 vs 3 | 24 vs 41 |
| **Alcohol consumption (drinkers vs non-drinkers)** | 1.03 (0.6-3.00) | 0.952 | 8 vs 8 | 89 vs 118 | 0.63 (0.22-1.86) | 0.406 | 6 vs 12 | 89 vs 118 |
| **Underweight (<18 kg/m^2^)** | 2.89 (0.32-25.80) | 0.342 | 1 vs 7 | 11 vs 126 | 1.94 (0.23-16.46) | 0.542 | 1 vs 8 | 11 vs 126 |
| **Overweight (26-30 kg/m^2^)** | 1.21 (0.38-3.88) | 0.737 | 5 vs 7 | 53 vs 126 | 1.43 0.45-4.59 | 0.545 | 5 vs 8 | 53 vs 126 |
| **Obesity and class 3 obesity (>30.1 kg/m^2^)** | 3.31 (0.67-16.41) | 0.142 | 2 vs 7 | 11 vs 126 | 4.59 (1.16-18.15) | **0.029** | 3 vs 8 | 11 vs 126 |
| **Family history of pancreatic cancer** | 0.75 (0.10-5.71) | 0.780 | 1 vs 15 | 21 vs 186 | 0.71 (0.09-5.38) | 0.74 | 1 vs 17 | 21 vs 186 |
| **Main duct vs branch duct** | - | - | 0 vs 15 | 0 vs 204 | - | - | 0 vs 16 | 0 vs 204 |
| **Mixed type vs branch duct** | 1.76 (0.21-14.61) | 0.600 | 1 vs 15 | 3 vs 204 | 4.73 (1.01-22.20) | 0.049 | 2 vs 16 | 3 vs 204 |
| **Type 2 diabetes** | 4.81 (1.70-13.63) | **0.003** | 6 vs 10 | 20 vs 187 | 1.78 (0.51-6.29) | 0.365 | 3 vs 15 | 20 vs 187 |
| ***WF and HRS*** |  |  |  |  |  |  |  |  |
| **Cyst size (mm)** | 1.20 (1.11-1.31) | **1.53×10^-5^** | 16 | 207 | 1.12 (1.04-1.20) | **0.002** | 18 | 207 |
| **Cyst size 15-29.9 mm vs <14.9mm** | 3.89 (1.06-14.28) | **0.040** | 13 vs 3 | 82 vs 125 | 1.87 (0.70-5.05) | **0.214** | 11 vs 7 | 82 vs 125 |
| **Cyst size >30 mm vs <14.9mm** | *-* | *-* |  |  | *-* | *-* |  |  |
| **Pancreatitis (acute and chronic pancreatitis)** | 3.06 (0.97-9.68) | 0.056 | 12 vs 4 | 17 vs 190 | 1.41 (0.31-6.31) | 0.655 | 2 vs 16 | 17 vs 190 |
| **Acute pancreatitis** | *-* | *-* |  |  | *-* | *-* |  |  |
| **Chronic pancreatitis** | 3.40 (1.06-10.88) | **0.039** | 4 vs 12 | 12 vs 193 | 1.68 (0.37-7.67) | 0.500 | 2 vs 16 | 12 vs 193 |

**>2WF/HRS** = analyses, no progression versus those with >2 WR/HRS features; **HRS** = analyses, no progression versus HRS alone. The analyses of BMI are all performed comparing each category with normal weight (18.5 -25 kg/m^2^). The association level of variables, cyst size >30mm and acute pancreatitis, were not calculated in the model “**No WF at diagnosis”** because are two worrisome features. **N. of prog** = the number of subjects that showed an IPMN progression, for the non-continuous variables are reported the number of analysed subjects Vs the number of subjects of the reference category; **N. of no prog = the** number of subjects that did not show an IPMN progression. All the analyses were adjusted by sex and age. The missing results are due to the absence of patients inside one of the groups defined for the analysis, making it impossible to obtain an association estimation.
